# Dimethyl fumarate attenuates reactive microglia and long-term memory deficits following systemic immune challenge

**DOI:** 10.1186/s12974-018-1125-5

**Published:** 2018-03-29

**Authors:** Hallel C. Paraiso, Ping-Chang Kuo, Eric T. Curfman, Haley J. Moon, Robert D. Sweazey, Jui-Hung Yen, Fen-Lei Chang, I-Chen Yu

**Affiliations:** 1 Department of Biology, Indiana University-Purdue University Fort Wayne, Fort Wayne, IN USA; 2 Department of Microbiology and Immunology, Indiana University School of Medicine, Fort Wayne, IN USA; 3 Department of Anatomy and Cell Biology, Indiana University School of Medicine, Fort Wayne, IN USA; 4 Department of Biochemistry and Molecular Biology, Indiana University School of Medicine, Fort Wayne, IN USA; 5 Department of Neurology, Indiana University School of Medicine, Fort Wayne, IN USA

**Keywords:** Microglia, Neuroinflammation, Dimethyl fumarate, Cognitive impairment, Astrocytes

## Abstract

**Background:**

Systemic inflammation is associated with increased cognitive decline and risk for Alzheimer’s disease. Microglia (MG) activated during systemic inflammation can cause exaggerated neuroinflammatory responses and trigger progressive neurodegeneration. Dimethyl fumarate (DMF) is a FDA-approved therapy for multiple sclerosis. The immunomodulatory and anti-oxidant properties of DMF prompted us to investigate whether DMF has translational potential for the treatment of cognitive impairment associated with systemic inflammation.

**Methods:**

Primary murine MG cultures were stimulated with lipopolysaccharide (LPS) in the absence or presence of DMF. MG cultured from nuclear factor (erythroid-derived 2)-like 2-deficient (*Nrf2*^*−/−*^) mice were used to examine mechanisms of DMF actions. Conditioned media generated from LPS-primed MG were used to treat hippocampal neuron cultures. Adult C57BL/6 and *Nrf2*^*−/−*^ mice were subjected to peripheral LPS challenge. Acute neuroinflammation, long-term memory function, and reactive astrogliosis were examined to assess therapeutic effects of DMF.

**Results:**

DMF suppressed inflammatory activation of MG induced by LPS. DMF suppressed NF-κB activity through Nrf2-depedent and Nrf2-independent mechanisms in MG. DMF treatment reduced MG-mediated toxicity towards neurons. DMF suppressed brain-derived inflammatory cytokines in mice following peripheral LPS challenge. The suppressive effect of DMF on neuroinflammation was blunted in *Nrf2*^*−/−*^ mice. Importantly, DMF treatment alleviated long-term memory deficits and sustained reactive astrogliosis induced by peripheral LPS challenge. DMF might mitigate neurotoxic astrocytes associated with neuroinflammation.

**Conclusions:**

DMF treatment might protect neurons against toxic microenvironments produced by reactive MG and astrocytes associated with systemic inflammation.

**Electronic supplementary material:**

The online version of this article (10.1186/s12974-018-1125-5) contains supplementary material, which is available to authorized users.

## Background

Dementia causes severe memory loss and destroys functional independence in affected individuals. Mounting evidence indicates that systemic inflammation acts as a significant driver of neurodegeneration and development of dementia [1]. Acute systemic inflammatory traumas not only cause profound cognitive dysfunction, such as delirium, but also lead to long-term cognitive decline and increase risk of developing dementia [2–4]. Low-grade peripheral inflammation chronically affects the volume of the hippocampus and increases risk of cognitive deficits in late life [5, 6]. During the progression of aging, the human body accumulates inflammatory attacks through repeated microbial infections, tissue injuries, or chronic disorders, such as obesity, diabetes, or atherosclerosis. Systemic inflammation caused by these inflammatory comorbidities can robustly alter brain inflammatory status through inducing phenotypic changes of the brain resident immune cells, microglia (MG).

MG play critical roles in maintaining tissue homeostasis of the brain. Upon stimulation, MG undergo a dynamic spectrum of activation patterns and change their functional properties [7–9]. Classically activated MG produce robust pro-inflammatory cytokines, including tumor necrosis α (TNFα), interleukin-1β (IL-1β), IL-12, and IL-6. The inflammatory activation of reactive MG contributes to the pathogenesis of neurodegeneration in various human neurological disorders [10]. Dysregulation in microglial activation leads to accumulations of “primed” MG that tend to react strongly to the stimuli from the periphery and get activated towards inflammatory phenotypes. Activated inflammatory MG trigger and orchestrate the process leading to chronic neuroinflammation and neurodegeneration [10].

Dimethyl fumarate (DMF) is a fumaric acid ester that is commonly used to treat psoriasis [11, 12]. The delayed-released DMF is approved as a therapy for the treatment of multiple sclerosis (MS) after showing its efficacy on reducing relapse rate and the number of new lesions in phase III clinical trials [13, 14]. DMF and its metabolites, monomethyl fumarate (MMF), exhibit immunomodulatory and anti-oxidant properties [15]. DMF stimulates anti-inflammatory responses and reduces oxidative stress by activating the nuclear factor (erythroid-derived 2)-like 2 (Nrf2), a transcription factor regulating anti-oxidant response pathways [16, 17]. Under normal conditions, Nrf2 is sequestered in the cytoplasm by Kelch ECH-associated protein 1 (Keap1) binding and is degraded through the ubiquitin-proteasome pathway. Application of DMF stabilizes Nrf2 and promotes its transcriptional activities. Nrf2 binds the antioxidant response elements (ARE) located within gene promoters and induces expressions of detoxification and anti-oxidant enzymes such as hemoxygenase-1 (HO-1), NADPH quinone oxidoreductase-1 (NQO1), glutamate-cysteine ligase, catalytic subunit (Gclc), and glutamate-cysteine ligase regulatory subunit (Gclm). However, the DMF mechanism of action is not fully understood. Nrf2-independent mechanisms may be involved in the therapeutic effects of DMF in MS [18, 19].

In chronic autoimmune disorders, such as MS, MG are thought to sustain the inflammatory responses that are initially triggered by infiltrating T cells from the periphery. Activated MG propagate neuroinflammation and drive disease evolution into the progressive phase, leading to irreversible demyelination and axonal loss [9, 20]. Reactive MG induced by systemic inflammation might result in progressive inflammation inside the brain, leading to neurodegeneration and subsequently cognitive impairment. In this study, we investigated the potential effects of DMF on modulating reactive MG associated with systemic inflammation. Our results demonstrate that DMF significantly suppresses acute neuroinflammation following peripheral lipopolysaccharide (LPS) challenge in mice. DMF treatment alleviates long-term memory deficits and mitigates reactive A1-like astrocytes associated with systemic inflammation.

## Methods

### Animals

C57BL/6, *Cx3cr1*^*gfp/+*^, and *Nrf2*^*−/−*^ mice were purchased from the Jackson Laboratory and bred at the animal facility of Indiana University School of Medicine, Fort Wayne. Mice were housed and maintained at 25 °C under a 12-h light/12-h dark cycle with ad libitum access to food and water. Adult female mice aged at 12–16 weeks were used for studies. All animal procedures in this study were conducted in accordance with the National Institutes of Health (NIH) Guide for the Care and Use of Laboratory Animals and approved by Purdue Animal Care and Use Committee.

### Reagents

Dimethyl fumarate (DMF) was purchased from Tocris Bioscience (Minneapolis, MN). Lipopolysaccharide (LPS; *Escherichia coli* O55:B5) and dimethyl sulfoxide (DMSO) were purchased from Sigma-Aldrich (St. Louis, MO). JC-1 dye (5,5′,6,6′-tetrachloro-1,1′,3,3′-tetraethylbenzimidazolylcarbocyanine iodide) was purchased from Thermo Fisher Scientific. Antibodies used for fluorescence-activated cell sorting (FACS) analysis were purchased from BioLegend (San Diego, CA): PE-conjugated anti-mouse CD11b (clone: M1/70) and CD86 (clone: PO3), PE/Cy7-conjugated anti-mouse CD80 (clone: 16-10A1), and APC-conjugated anti-mouse CD40 (clone: 3/23). For the western blot analysis, rabbit anti-Nrf2 and anti-HO-1 antibodies were purchased from Proteintech (Chicago, IL). Rabbit anti-phospho-IKK-α/β and rabbit anti-inducible nitric oxide synthase (iNOS) antibodies were purchased from Cell Signaling (Danvers, MA). For immunostaining of astrocytes, mouse anti-glial fibrillary acidic protein (GFAP) antibody was purchased from Cell Signaling (Danvers, MA); rabbit anti-aquaporin-4 (Aqp4) antibody was purchased from Sigma-Aldrich. Recombinant mouse TNFα and IL-1α were purchased from BioLegend (San Diego, CA).

#### Cell culture and drug treatment

Primary MG cells were obtained as previously described with some modifications [21]. Briefly, cells were cultured in medium DMEM/F12 containing 10% heated-inactivated fetal bovine serum, 2 mM l-glutamine, and 1X antibiotic/antimycotic. The medium was supplemented with 5 ng/ml granulocyte macrophage-colony stimulating factor (GM-CSF) on days in vitro (DIV) 5 and 10 after plating. On DIV 15, MG were harvested by shaking the flasks. The purity of MG cells was examined using FACS analysis. More than 95% of harvested cells were positive for CD11b expression (Additional file 1: Figure S1A). MG cells were seeded at desired densities and pre-treated with DMF (0, 10, and 100 μM in culture medium containing 0.001% DMSO final concentration) for 1 h and then stimulated with 100 ng/ml LPS for various times as indicated in the text and/or figure legends. The working concentration of LPS was prepared from the stock using culture medium as the diluent. Enriched astrocyte cultures were prepared as previously described [22]. Briefly, cells were harvested and maintained in the same medium for primary MG in the absence of GM-CSF. On DIV 8, MG and oligodendrocyte precursor cells growing on the top of confluent astrocyte monolayer were detached by shaking at 250 rpm for 6 h. The astrocyte layer was detached using trypsin EDTA and re-plated to cell culture flasks. On DIV 20, enriched astrocytes were prepared and seeded at desire density for experiments. The purity of enriched astrocyte cultures was examined using the GFAP and Aqp4 immunostaining analysis, showing 70–75% of cells are GFAP+ and Aqp4+ astrocytes (Additional file 1: Figure S1C). The astrocyte cultures were pre-treated with DMF (0 and 100 μM) for 1 h followed by TNFα (30 ng/ml) and IL-1α (3 ng/ml) stimulation for 24 h as indicated.

#### RNA isolation and real-time PCR

Total RNAs were isolated from cells or tissues using RNeasy RNA isolation kit (Qiagen). cDNAs were prepared by reverse transcription using High-Capacity cDNA Reverse Transcription Kit with random primers (Life Technologies, CA). Quantitative real-time PCR (qPCR) was performed using TaqMan gene expression probes and PCR Master Mix (Applied Biosystems, Life Technologies, CA).

#### Immunocytochemistry and confocal microscopy

Primary MG were treated as described. After treatment, cells were fixed in 2% paraformaldehyde following with phosphate-buffered saline (PBS) wash. Cells were permeabilized and blocked with 3% normal goat serum for 30 min at room temperature (RT). Cells were incubated with primary antibodies, rabbit anti-Iba1 (WAKO, Richmond, VA), rabbit anti-NF-κB p65 or anti-cleaved Caspase-3 (Cell Signaling, Danvers, MA) for 1 h at RT. After washing, cells were incubated with Alexa Fluor 594-conjugated goat anti-rabbit (Life Technologies, CA) for 1 h at RT. After washing, cell nuclei were counterstained with Hoechst 33342 (Life Technologies, CA). Images were acquired on Fluoview FV10i confocal microscope with an × 60 objective using the FV10iO software (Olympus) and analyzed using ImageJ, NIH.

#### Western blotting

Cell lysates were prepared with cold RIPA buffer (50 mM Tris-HCl, pH 8.0, 150 mM NaCl, 1% NP-40, 0.5% sodium deoxycholate, 0.15% SDS, 1 mM phenylmethylsulfonyl fluoride, phosphatase inhibitor, and protease inhibitor cocktail). Proteins were resolved in 10% SDS-PAGE and transferred to polyvinylidene difluoride membranes (Millipore). The membrane was blocked and incubated with specific primary antibodies. After incubating with secondary antibodies, the blots were detected using ImmobilonTM Western Chemiluminescent HRP Substrate (EMD Millipore, MA).

#### Analysis of LPS-induced neuroinflammation after DMF treatment

Adult *Cx3cr1*^*gfp/+*^ mice were randomly assigned to different treatment groups and intraperitoneally (ip) injected with 0.9% saline or LPS (1 mg/Kg). For the DMF-treated group, mice were administered DMF (45 mg/Kg, ip) at 1 h before and 8 h after LPS injection [23, 24]. The ip administration route was performed to achieve higher bioavailability of DMF during shorter period of time after dosing and to decrease the potential stress to the sick LPS-treated mice. The brains were harvested 24 h post-injection. For imaging analysis, the harvested brains were fixed with 4% paraformaldehyde and cryoprotected with 30% sucrose. Serial 15-μm frozen sections were cut in a cryostat, mounted on superfrost plus slides, and stored at − 20 °C until use. Brain slides were prepared with ProLong Gold anti-fade mountant (Invitrogen). The fluorescence microscope BX53 (Olympus, PA), Nuance FX multispectral imaging system, and Nuance software (PerkinElmer) were used for image acquisition and analysis. For FACS analysis, brain mononuclear cells were isolated as previously described [21, 25]. The isolated cells were incubated with an antibody cocktail containing anti-CD86, CD80, and CD40. After staining, cells were subjected to FACS analysis. Mononuclear cell populations, including MG and infiltrating immune cells, were gated as GFP+ cells. For analysis of pro-inflammatory cytokines, the harvested brains were homogenized and subjected to RNA isolation. The expression levels of inflammatory mediators were measured using qPCR with interested TaqMan gene expression probes.

#### Cell viability assay

Primary MG were treated with DMF (0, 10, and 100 μM) for 1 h followed by LPS (1 μg/ml) stimulation. To produce LPS/DMF-free conditioned-medium (CM), the culture medium was changed to neurobasal medium (Life Technologies, CA) containing 2 mM l-glutamine and 1 × N2 supplement (Life Technologies, CA) at 6 h after LPS stimulation to wash out LPS and DMF. The microglial CM was collected 24 h after LPS. The microglial CM was applied to differentiate HT22 hippocampal neurons. Mouse HT22 hippocampal neurons were differentiated as previously described before exposed to microglial CM [21]. At 24 h after incubating with microglial CM, the viability of neurons was examined using a 3-[4,5-dimethylthiazol-2-yl]-2,5-diphenyltetrazolium bromide (MTT) assay.

#### Mitochondrial membrane potential measurement

The mitochondrial membrane potential was detected using fluorescent probe JC-1. Cells were incubated with 10 μg/ml of JC-1 dye for 10 min at 37 °C followed by PBS wash. Healthy cells with polarized mitochondria predominantly exhibited orange-red fluorescence due to an aggregated form of JC-1 (Ex 535 nm, Em 590 nm). When cells contained depolarized mitochondria, they displayed green fluorescence reflected by a monomeric form of JC-1 (Ex 485 nm, Em 530 nm). Six to eight fields from each treatment group in an experiment were acquired using inverted fluorescence microscope DMI6000B with an attached digital camera (Leica Microsystems, IL).

#### Immunohistochemistry

The mouse brains were harvested and processed to prepare brain sections as previously described [21, 25]. Brain sections were blocked and incubated with primary antibodies, rabbit anti-Iba1 (1:2500, WAKO, VA), or rabbit anti-GFAP (1:3000, Cell Signaling, Danvers, MA) for 2 h at RT. After the secondary antibody incubation, slides were developed with 3,3′-diaminobenzidine (DAB) liquid substrate (DAKO, CA). Then, slides were counterstained with Mayer’s hematoxylin (Sigma-Aldrich) and mounted with Permount (FisherSci). Two brain sections from each mouse were stained and analyzed. Stained slides were imaged (microscope: BX53, camera: DP72, Olympus) and analyzed using CellSens software (Olympus) by an experimenter blinded to experimental conditions.

#### Novel object recognition test

The test was performed using a square arena (50 cm × 50 cm × 34.5 cm). Adult C57BL/6J mice were randomly assigned to different treatment groups. Mice were handled for 3 days for habituation of handling. Mice were then acclimated to the arena over 3 days, one 10-min trial per day. Mice were subjected to a single-dose LPS (1 mg/Kg, ip) injection. DMF (30 mg/Kg, ip) was administered every other day for 3 days as indicated. The changes of body weight were measured daily to monitor mice recovering from sickness following LPS injection. Four days after LPS injection, mice were exposed to the same arena containing two identical objects (henceforth “familiar” object) for 10 min, as the acquisition session. Twenty-four hours after the acquisition, the recognition session was performed by replacing one of the familiar objects with a novel object. Any-maze software (Stoelting, IL) was used to track and analyze the movements of the mice. The behavioral experiments were performed by researchers blinded to treatment groups. Results were expressed as percentage of time exploring each object.

### Statistical analysis

Data are presented as mean ± SEM. For in vivo studies with the groups containing ≥ 7 mice, a normal distribution of the data was confirmed by Shapiro-Wilk normality test. Comparisons between two groups were done using the unpaired *t* test, whereas comparisons between multiple groups were done by one-way ANOVA followed by Bonferroni post hoc test using GraphPad Prism software. Statistical significance was determined with *p* values < 0.05.

## Results

### DMF suppresses inflammatory activation of MG

**Fig. 1:**
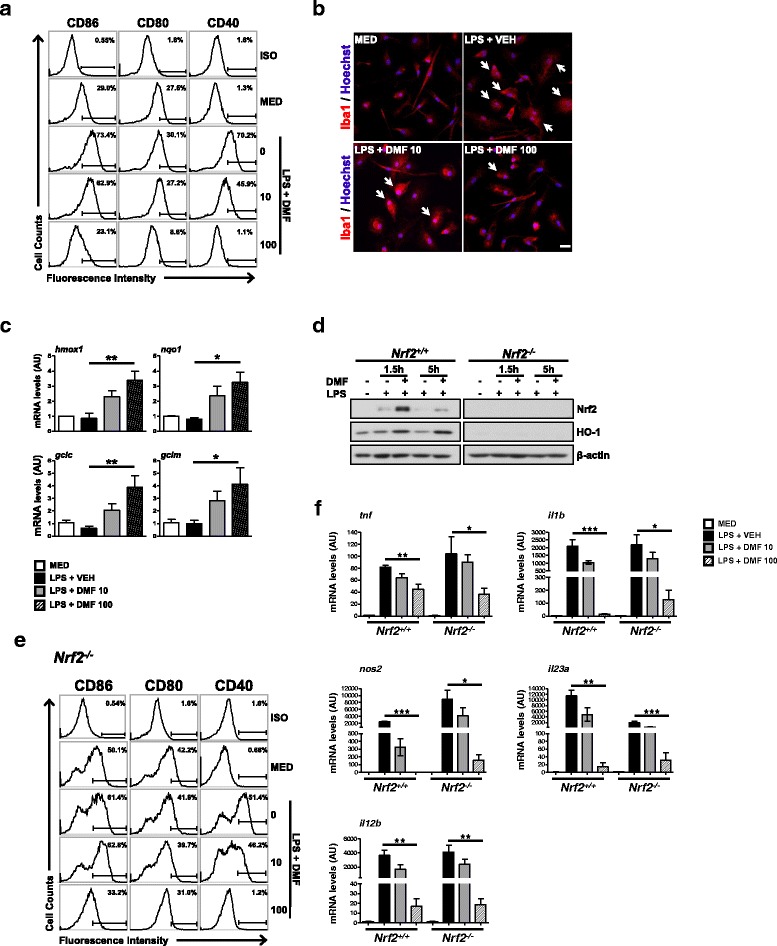
DMF suppresses LPS-induced reactive MG. Primary MG were stimulated by LPS (100 ng/ml) in the absence or presence of DMF for various time periods. **a** Microglial surface markers, CD86, CD80, and CD40, were examined at 18 h after stimulation using FACS analysis. Representative flow plots from five independent experiments are shown. ISO, the isotype control antibody. MED, cell culture medium. **b** Morphological changes of MG at 18 h after stimulation were examined using Iba1 immunostaining. Arrows indicate activated MG with increased expression of Iba1 and enlarged cell bodies. Cell nuclei were stained blue with Hoechst 33342. Scale bar, 20 μm. **c** The expressions of DMF-induced Nrf2 target genes were examined at 3 h after stimulation. The mRNA levels of *hmox1*, *nqo1*, *gclc*, and *gclm* were quantified using qPCR. Data presented are from 3 to 5 independent experiments. *p < 0.05, **p < 0.01, by one-way ANOVA with Bonferroni’s post hoc multiple comparison test. **d** The *Nrf2*^*+/+*^ and *Nrf2*^*−/−*^ microglial cells were harvested for western blot analysis to examine Nrf2 and HO-1 proteins at 1.5 and 5 h after stimulation. Representative results are shown from three independent experiments. **e** The CD86, CD80, and CD40 expressed on the surface of *Nrf2*^*−/−*^ MG were examined at 18 h after stimulation using FACS analysis. Representative flow plots from three independent experiments are shown. **f** Expressions of inflammatory mediators were examined in *Nrf2*^*+/+*^ and *Nrf2*^*−/−*^ MG at 3 h after stimulation. The mRNA levels of *tnf*, *il1b*, *nos2*, *il23a*, and *il12b* in treated MG were quantified using qPCR. Data presented are from three independent experiments. *p < 0.05, **p < 0.01, ***p < 0.001, by one-way ANOVA with Bonferroni’s post hoc multiple comparison test.

We first asked whether DMF is capable of modulating phenotypes of MG in response to LPS stimulation. We examined expressions of maturation markers, CD86, CD80, and CD40, on MG cells stimulated with LPS in the absence or presence of DMF. The LPS stimulation induced microglial activation resulting in the increase of these surface markers, whereas DMF treatment significantly suppressed these markers in a dose-dependent manner (Fig. 1a and Additional file 2: Figure S2A). In addition, DMF was able to inhibit morphological changes and Iba1 upregulation in MG stimulated with LPS (Fig. 1b). The concentrations of DMF used in these studies did not affect the cell viability of MG, suggesting that the suppressive effect of DMF on MG is not mediated through cytotoxicity (Additional file 1: Figure S1B).

DMF is shown as a strong inducer of Nrf2 in animal models of MS [16, 17]. We also found that the expressions of Nrf2 target genes were significantly upregulated in MG treated with DMF (Fig. 1c). To investigate whether Nrf2 mediates actions of DMF in MG, we compared effects of DMF on *Nrf2*^*+/+*^ and *Nrf2*^*−/−*^ MG stimulated with LPS. In *Nrf2*^*+/+*^ MG, LPS stimulation slightly increased the level of Nrf2 protein, but not its downstream target, HO-1 (Fig. 1d). The DMF treatment significantly upregulated the protein levels of Nrf2 and HO-1. In contrast, *Nrf2*^*−/−*^ MG did not exhibit induction of Nrf2 nor HO-1, after being treated with DMF, demonstrating the deficiency of Nrf2. We next analyzed the effects of DMF on *Nrf2*^*−/−*^ MG stimulated with LPS. The DMF suppressed the induction of surface markers in *Nrf2*^*−/−*^ MG after LPS stimulation, similar to its action on *Nrf2*^*+/+*^ MG (Fig. 1e and Additional file 2: Figure S2B). These results indicate that DMF might act on multiple pathways to modulate microglial phenotype in response to LPS stimulation.

The release of pro-inflammatory cytokines indicates activated MG moving towards an inflammatory phenotype. Further analyzing expressions of cytokines, we found that DMF suppressed LPS-induced mRNA expressions of TNF-α, IL-1β, iNOS, IL-23p19, and IL-12p40 in *Nrf2*^*+/+*^ MG (Fig. 1f). DMF was capable of suppressing the induction of inflammatory mediators in *Nrf2*^*−/−*^ MG stimulated with LPS. We noticed that the suppressive effect of DMF on some of the inflammatory mediators in *Nrf2*^*−/−*^ MG was not as powerful as its suppressive action in *Nrf2*^*+/+*^ MG. Taken together, these results suggest that DMF suppresses inflammatory activation of reactive MG induced by LPS potentially through both Nrf2-dependent and Nrf2-independent mechanisms.

### DMF inhibits LPS-induced NF-κB activation in MG

**Fig. 2:**
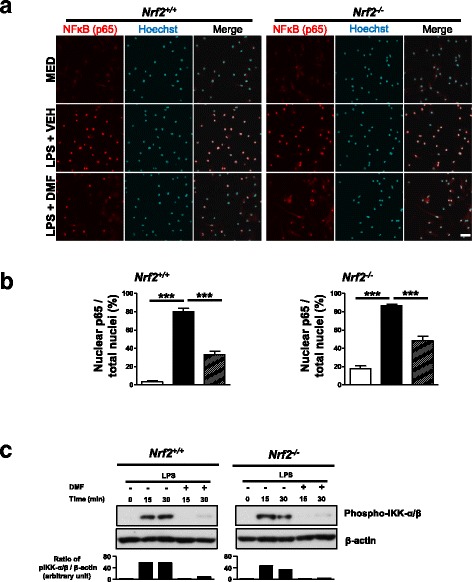
DMF acts on Nrf2-independent mechanisms to suppress NF-κB activation induced by LPS. Primary *Nrf2*^*+/+*^ and *Nrf2*^*−/−*^ MG were stimulated with LPS (100 ng/ml) in the absence or presence of DMF (100 μM) for various time periods. **a** Nuclear translocation of NF-κB (p65) was examined at 15 min after stimulation. Representative images of p65 immunostaining from three independent experiments are shown. Hoechst 33342-stained cell nuclei are depicted in blue. MED, cell culture medium. VEH, vehicle control. Scale bar, 20 μm. **b** The percentage of p65-positive nuclei/total cell nuclei in each treatment group was quantified. Data presented are from three independent experiments. ***p < 0.001, by one-way ANOVA with Bonferroni’s post hoc multiple comparison test. **c** The western blot analysis of phospho-IKKα/β at 15 or 30 min after stimulation. Data presented are representative blots from three independent experiments

NF-κB regulates the transcription of a variety of pro-inflammatory genes in different immune cells. To test whether DMF acts on NF-κB signaling pathway to regulate microglial phenotypes, we examined the nuclear translocation of NF-κB p65 induced by LPS in the absence or presence of DMF. When stimulated with LPS, about 80% of MG cells exhibited nuclear p65 immunoreactivity (Fig. 2a, b). DMF treatment significantly suppressed nuclear translocation of p65 in both *Nrf2*^*+/+*^ and *Nrf2*^*−/−*^ MG stimulated with LPS. Notably, *Nrf2*^*−/−*^ MG exhibited a higher level of p65 nuclear translocation at the resting state and upon LPS stimulation, suggesting that Nrf2 deficiency might promote inflammatory phenotypes of MG. In addition, DMF abolished LPS-induced phosphorylation of IKK-α/β in both *Nrf2*^*+/+*^ and *Nrf2*^*−/−*^ MG (Fig. 2c). Taken together, these results demonstrate the Nrf2-independent mechanisms mediating actions of DMF to suppress LPS-induced NF-κB activity in MG.

### DMF reduces MG-mediated neurotoxicity

**Fig. 3:**
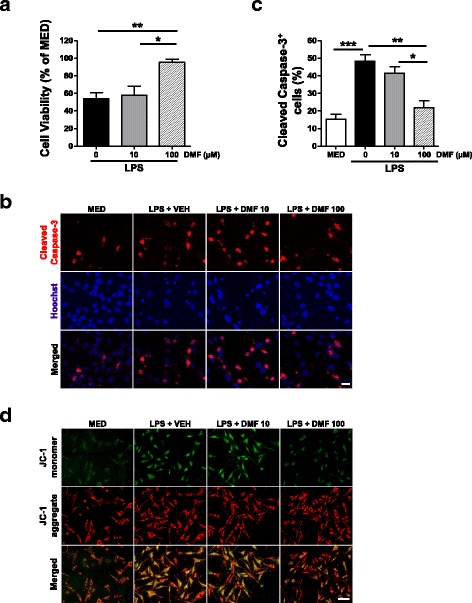
DMF suppresses MG-mediated neurotoxicity. MG were stimulated with LPS (1 μg/ml) in the absence or presence of DMF. Microglial CM was collected and applied to cultured hippocampal neurons. Neurons were exposed to microglial CM for 24 h. **a** The cell viability was assessed by MTT assay. Data presented are from four independent experiments. *p < 0.05, **p < 0.01, by one-way ANOVA with Bonferroni’s post hoc multiple comparison test. **b** The Caspase-3 activation was examined by immunostaining to detect cell death in neurons. Cell nuclei were stained blue with Hoechst 33342. Representative images are from three independent experiments. MED, cell culture medium. VEH, vehicle control. Scale bar, 20 μm. **c** The cleaved Caspas-3^+^ cells were quantified and compared with total cells (Hoechst-stained cells). Data presented are percentage of cleaved Caspas-3^+^ cells in each treatment group. Results presented are from three independent experiments. *p < 0.05, **p < 0.01, ***p < 0.001, by one-way ANOVA with Bonferroni’s post hoc multiple comparison test. **d** Representative images show a shift of JC-1 fluorescence from orange red (mitochondrial membrane polarized) to green (mitochondrial membrane depolarized) in neurons incubated with CM from LPS-primed MG for 6 h. Neurons incubated with the CM generated from DMF-treated MG (100 μM) show decreased JC-1 green fluorescence. MED, cell culture medium. VEH, vehicle control. Scale bar, 40 μm. Results are from three independent experiments

We next asked whether manipulating microglial phenotypes and functions by DMF can foster neuroprotection. We exposed differentiated HT22 hippocampal neurons to CM derived from MG stimulated with LPS in the absence or presence of DMF. After 24 h of exposure, there was a substantial reduction in cell viability of neurons incubated with CM generated from LPS-primed MG (Fig. 3a). The induction of inflammatory phenotype in MG was examined after collecting CM, showing increased iNOS protein in MG stimulated with LPS (Additional file 2: Figure S2C). The DMF treatment was able to suppress the LPS-induced iNOS in MG. When MG were stimulated with LPS in the presence of DMF, they generated less toxic CM, resulting in more survived neurons after incubation. In consistent with neuronal cell viability, neurons exposed to CM derived from LPS-primed MG showed increased Caspase-3 activation, indicating increased neuronal cell death induced by microglial CM (Fig. 3b, c). In contrast, the CM generated from DMF-treated MG did not induce significant Caspase-3 activation in neurons compared to the vehicle-treated controls.

Mitochondrial dysfunction plays a critical role in the degeneration and death of neurons [26]. We monitored mitochondrial health status using molecular probe JC-1 to distinguish energized (high membrane potential) and de-energized mitochondria (membrane depolarization) in neurons after short-term (6 h) exposure to microglial CM (Fig. 3d). Incubating neurons with CM generated from LPS-primed MG resulted in a shift of JC-1 fluorescence emission from orange red (JC-1 aggregates) to green (JC-1 monomer). These data indicate that CM from LPS-primed MG leads to disrupted membrane potential of mitochondria in neurons. In contrast, the CM generated from DMF-treated MG did not significantly cause mitochondrial abnormalities in neurons. Taken together, our results demonstrate that DMF might protect neurons from inflammatory insults by modulating toxic factors released from reactive MG associated with inflammation.

### DMF suppresses LPS-induced acute neuroinflammation

**Fig. 4:**
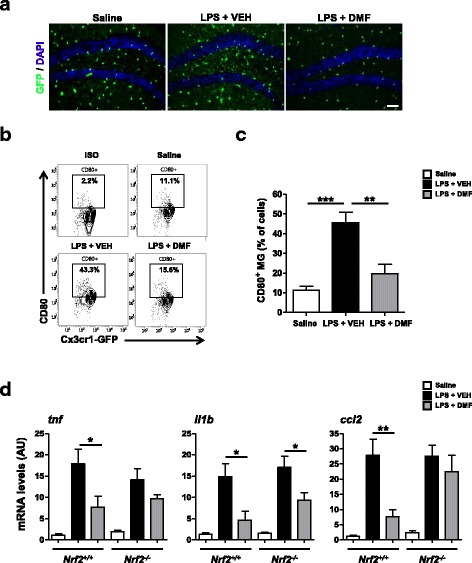
DMF reduces LPS-induced acute neuroinflammation. *Cx3cr1*^*gfp/+*^ mice were treated with a single-dose LPS injection (1 mg/Kg, ip). DMF (45 mg/Kg) was administered through ip injection. At 24 h after LPS injection, the mouse brains were harvested and subjected to analyses. **a** Representative images show GFP^+^ cells in the hippocampus of *Cx3cr1*^*gfp/+*^ mice that received saline, LPS + vehicle (VEH), or LPS + DMF treatment. *N* = 7 mice/group. Cell nuclei were stained with DAPI (blue). Scale bar, 40 μm. **b** Mononuclear cells were isolated from the brain homogenates and subjected to FACS analysis. The levels of CD80 marker on gated GFP^+^ cell populations were examined. Representative flow plots from three treatment groups, saline, LPS + VEH, LPS + DMF are shown. *N* = 7 mice/group. ISO, the isotype control antibody. **c** The proportion of CD80^+^ cells was determined. *N* = 7 mice/group. **p < 0.01, ***p < 0.001, by one-way ANOVA with Bonferroni’s post hoc multiple comparison test. **d** Effects of DMF treatment on inflammatory mediators induced by LPS injection in *Nrf2*^*+/+*^ (*Cx3cr1*^*gfp/+*^) or *Nrf2*^*−/−*^ mice. The mRNA levels of pro-inflammatory cytokines or chemokine (*tnf*, *il1b*, or *ccl2*) in the brain were measured using qPCR. *N* = 7 mice/group for *Nrf2*^*+/+*^ mice. *N* = 5–6 mice/group for *Nrf2*^*−/−*^ mice. *p < 0.05, **p < 0.01, by one-way ANOVA with Bonferroni’s post hoc multiple comparison test.

To investigate whether DMF modulates microglial activation in vivo, we treated *Cx3cr1*^*gfp/+*^ mice with a single dose of LPS injection (1 mg/Kg, ip) and examined the brain 24 h post-injection. The LPS injection led to increased GFP^+^ cells in the hippocampus of the brain, indicating acute neuroinflammation in response to LPS challenge (Fig. 4a). The administration of DMF was able to reduce numbers and morphological changes of GFP^+^ cells in the hippocampus. We further isolated brain mononuclear cells and examined maturation markers of GFP^+^ cells, which include activated MG and potentially infiltrating peripheral immune cells, such as monocytes/macrophages, following LPS challenge. The LPS challenge significantly upregulated CD80 expression on GFP^+^ cells, whereas DMF treatment reduced CD80 upregulation induced by LPS challenge (Fig. 4b, c). The expressions of CD86 and CD40 on the surface of GFP^+^ cells were not increased, while examined at 24 h after LPS challenge (data not shown). In addition, DMF treatment significantly suppressed de novo cytokine production within the brain following peripheral LPS challenge (Fig. 4d). Intriguingly, the suppressive effect of DMF on brain-derived inflammatory cytokine was blunted in LPS-challenged *Nrf2*^*−/−*^ mice. The suppression of IL-1β and TNFα mRNAs by DMF treatment was partially retained in *Nrf2*^*−/−*^ mice. However, the suppression of Ccl2 mRNA by DMF treatment was not observed in *Nrf2*^*−/−*^ mice. These data indicate that the Nrf2-dependent mechanism mediates the actions of DMF to suppress acute neuroinflammation induced by LPS. Taken together, these results suggest that DMF treatment alleviates acute inflammatory responses in the brain induced by peripheral LPS challenge.

### DMF alleviates cognitive deficits induced by systemic immune challenge

**Fig. 5:**
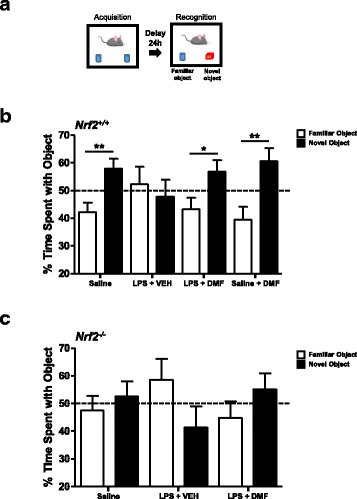
DMF treatment alleviates cognitive deficits following systemic immune challenge. **a** This figure illustrates the novel object recognition task using a 24-h retention interval. **b** Adult C57BL/6 mice were subjected to saline or LPS injection. DMF or vehicle (VEH) treatment was administered every other day for 3 days following LPS injection. At 5 days after LPS injection, the mice were subjected to the novel object recognition task. The time that mice spent with familiar or novel object was measured. Percentage of exploration time on the object was calculated. *N* = 10–16 mice/group. *p < 0.05, **p < 0.01, compared to familiar object by unpaired Student’s *t* test. Data of saline, LPS + VEH, and LPS + DMF treatment are from three independent experiments; data of saline + DMF treatment are from two independent experiments. **c** Adult *Nrf2*^*−/−*^ mice were subjected to saline, LPS + VEH, or LPS + DMF injection as described. At 5 days after LPS injection, the mice were subjected to the novel object recognition task. The time that mice spent with familiar or novel object was measured. Percentage of exploration time on the object was calculated. *N* = 8–12 mice/group. There was no statistical significance between the familial and the novel object when data were analyzed by unpaired Student’s *t* test. Data presented are from two independent experiments

We further investigated whether DMF treatment can alleviate the impairment of cognition driven by systemic inflammation. We treated mice with a single dose of LPS (1 mg/Kg, ip) and administered three doses of DMF (30 mg/Kg, ip) as shown in Additional file 3: Figure S3A. The changes of body weight were monitored daily to ensure that mice recovered from transient sickness after LPS injection (Additional file 3: Figure S3B). At 5 days after LPS challenge, mice were subjected to the novel object recognition task (Fig. 5a). The 24-h delayed paradigm was used to access the long-term memory function [27]. Mice that received saline injection showed a clear preference for the novel object, but mice that received LPS injection did not (Fig. 5b). The total exploratory or locomotor activity in mice was not affected at the time when tasks were performed (Additional file 3: Figure S3C). These results show that LPS-treated mice were not able to distinguish between the novel and familiar objects, indicating an impairment in recognition memory. In contrast, mice that received DMF treatment following LPS challenge exhibited a preference towards the novel object, suggesting that DMF treatment rescues the long-term memory deficits caused by systemic immune challenge.

Moreover, we examined *Nrf2*^*−/−*^ mice that were systemically challenged with LPS and subjected to novel object recognition task. We observed exacerbation of sickness with significant weight loss and increased mortality rate (about 20–30%) following LPS challenge in *Nrf2*^*−/−*^ mice (Additional file 3: Figure S3D), suggesting that Nrf2 plays an important role in alleviating systemic inflammation. The *Nrf2*^*−/−*^ mice exhibited the impairment in recognition memory regardless of LPS challenge (Fig. 5c and Additional file 3: Figure S3E). The administration of DMF, although was not able to fully rescue the memory deficits, tended to mitigate effects of LPS challenge. These results suggest that Nrf2 dysfunction might alter the cognitive function of the brain.

### DMF suppresses chronic neuroinflammation associated with systemic immune challenge

**Fig. 6:**
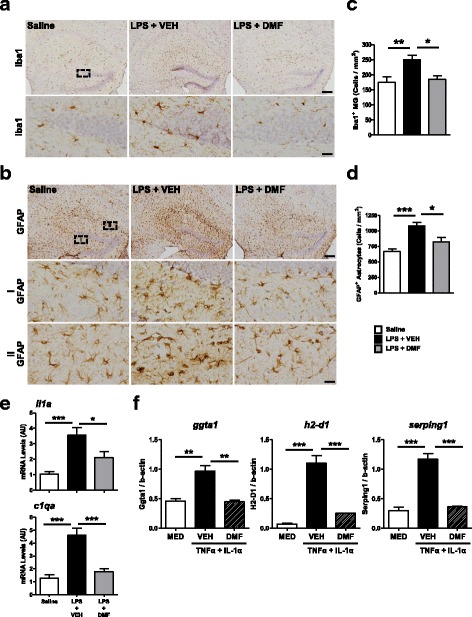
DMF alleviates reactive astrogliosis following systemic immune challenge. **a** Iba1^+^ MG and **b** GFAP^+^ astrocytes were examined by immunohistochemistry at 2 weeks after a single-dose LPS injection. DMF was administered every other day for 3 days following LPS injection. Representative images show the hippocampus of mice from three treatment groups, saline, LPS + VEH, LPS + DMF. *N* = 4–5 mice/group. High magnification images of circled areas are shown at the lower panel. Quantification results of **c** Iba1^+^ MG and **d** GFAP^+^ astrocytes are shown. *p < 0.05, **p < 0.01, and ***p < 0.001 by one-way ANOVA with Bonferroni’s post hoc multiple comparison test. Scale bar, 200 μm (low magnification) or 20 μm (high magnification). **e** The mRNA levels of inflammatory molecules, *il1a* and *c1qa*, in the brain were quantified using qPCR. *N* = 7 mice/group. *p < 0.05, ***p < 0.001, by one-way ANOVA with Bonferroni’s post hoc multiple comparison test. **f** Enriched astrocyte cultures were stimulated with TNFα (30 ng/ml) and IL-1α (3 ng/ml) in the absence or presence of DMF. The expressions of A1 signature genes were measured at 24 h after stimulation. The mRNA levels of *ggta1*, *h2-d1*, and *serping1* were quantified using qPCR. Data presented are from three independent experiments. **p < 0.01, ***p < 0.001, by one-way ANOVA with Bonferroni’s post hoc multiple comparison test.

The systemic immune responses, although being resolved, still led to a long-lasting impact to the brain. There was a slight increase of Iba1-expressing MG in the hippocampus of LPS-treated mice, compared to saline- or DMF-treated mice, even 2 weeks after LPS injection (Fig. 6a, c). It is noteworthy that we observed profound GFAP^+^ astrocytes in the hippocampus of LPS-treated mice compared to saline-injected mice (Fig. 6b, d). The GFAP^+^ astrocytes were significantly reduced in the hippocampus of DMF-treated mice. These results indicate that systemic immune challenge triggers robust but sustained responses in astrocytes and that DMF administration alleviates reactive astrocytes in response to inflammation. A recent study shows that TNFα, IL-1α, and C1q released from inflammatory MG transform astrocytes towards neurotoxic A1 astrocytes [28]. We analyzed whether DMF modulates expressions of IL-1α and C1q in the brain following peripheral LPS challenge. In addition to TNFα, DMF treatment significantly reduced the expressions of IL-1α and C1q in the brain induced by LPS injection (Fig. 6e). We further tested whether DMF mitigates A1 phenotype in primary astrocytes. In vitro, TNFα and IL-1α were able to induce A1 astrocyte specific genes, including g*gta1*, h*2-d1*, and *serping1*. The expression of these genes was suppressed in DMF-treated astrocytes (Fig. 6f). Taken altogether, these results suggest that the protective effect of DMF on rescuing neuronal dysfunction in LPS-challenged mice might potentially be mediated through its suppressive effect on the generation of neurotoxic A1 astrocytes.

## Discussion

In the present study, we investigated the translational potential of DMF for the treatment of cognitive impairment associated with systemic inflammation. DMF exerts distinct neuroprotective effects and clinical efficacy for the treatment of relapsing-remitting MS [13, 14]. Here, we report a direct modulatory effect of DMF on inflammatory MG induced by LPS. DMF effectively suppresses maturation markers, morphological changes, and production of inflammatory mediators in cultured MG. In line with our results, several studies also report that DMF shows effective suppression on LPS-induced production of inflammatory cytokines in human fetal and adult MG cultures and in murine MG cultures [29, 30].

As a consequence of inflammatory activation, the toxic factor released from reactive MG is able to induce neurotoxicity [31]. We show that the CM from LPS-primed MG induces Caspase-3 activation in neurons after 24 h of exposure, while shorter time of exposure (6 h) results in disrupted mitochondrial membrane potential in neurons. DMF reduces inflammatory phenotype of MG and ameliorates MG-mediated neurotoxicity. Similar to our results, DMF is reported to reduce neuronal dysfunction through mitigating inflammatory activation of MG induced by LPS [32]. MMF is shown to preserve neuronal viability through modulating microglial phenotype stimulated with virus-infected monocytes [33].

In addition to in vitro studies, we identify that DMF reduces acute neuroinflammation following peripheral LPS challenge in mice. DMF administration reduces morphological changes and maturation marker induction in brain-associated immune cells, including resident and infiltrating immune cells, following LPS challenge. Given that DMF is reported to suppress M1 polarization of monocytes/macrophages in different disease models, we speculate that the reduced neuroinflammation in LPS/DMF-treated mice could be contributed by the actions of DMF on peripheral immune cells [19, 34]. LPS is a strong activator of Toll-like receptor 4 signaling, which induces inflammatory mediators robustly. Excessive pro-inflammatory cytokines in the brain can impair neuronal viability and lead to cognitive dysfunction [35–39]. DMF administration significantly suppresses the expression of pro-inflammatory cytokines in the brain following peripheral LPS challenge, supporting its potential to ameliorate cognitive dysfunction associated with systemic inflammation [40, 41]. Our results provide promising evidence that DMF treatment rescues deficits in long-term recognition memory affected by systemic inflammation.

Several studies have reported that Nrf2-independent mechanisms mediate the action of DMF [19, 32, 42, 43]. In this study, we also observe that Nrf2-independent actions of DMF modulate inflammatory MG and neuroinflammation in mice following LPS challenge. However, we acknowledge that the suppressive effect of DMF on LPS-induced neuroinflammation is blunted in *Nrf2*^*−/−*^ mice. Nrf2 is reported exhibiting immunomodulatory and anti-inflammatory activities in T cells [44]. The nuclear factor NF-κB is a key transcription factor regulating immune responses. It is known that there is a cross-talk between Nrf2 and NF-κB signaling pathways [45]. Activation of Nrf2 attenuates NF-κB activity, which is possibly mediated by HO-1-derived bilirubin in endothelial cells [46]. Nrf2 knockdown is associated with increased MafK activity, which positively promotes p65 acetylation and its subsequent transactivation activity in hepatocytes [47]. Our findings provide the evidence of cross-talk between Nrf2 and NF-κB signaling pathways in MG. The deficiency of Nrf2 in MG results in a higher level of p65 nuclear translocation at the resting state. This result suggests potential role of Nrf2 in regulating the intrinsic homeostasis of MG. Moreover, *Nrf2*^*−/−*^ mice show exaggerated sickness response to LPS challenge, demonstrating the important role of Nrf2-mediated anti-inflammatory responses.

When examining long-term effects of peripheral LPS challenge to the brain, we observed profound reactive astrocytes in the hippocampus of LPS-treated mice. It is noteworthy that astrocytes are able to produce chemokine Ccl2 in response to cytokine stimulation in animal models. The astrocyte-derived chemokine Ccl2 is found to recruit circulating Ccr2^+^ monocytes infiltrating into the brain, which exacerbates the degeneration of neurons [48, 49]. DMF administration significantly suppresses transcription of Ccl2 mRNA in the brain induced by LPS challenge. Our results suggest potential modulatory effects of DMF on astrocytes. In line with our findings, DMF, but not MMF, is shown to reduce the secretion of Ccl2 from cultured astrocytes stimulated with pro-inflammatory cytokines [50]. Moreover, we observed that DMF inhibits expressions of essential molecules required for the generation of neurotoxic A1 astrocytes, including TNFα, IL-1α, and C1q, in the mouse brain following LPS challenge. Our subsequent examinations showed that the induction of A1 signature genes is suppressed by DMF treatment in cultured astrocytes, suggesting the action of DMF-regulating astrocyte phenotypes. Genetic over-expression of Nrf2 in astrocytes is reported to rescue neurodegeneration in the amyotrophic lateral sclerosis (ALS) animal model [51]. It would require further investigation to examine the potential of DMF regulating phenotypic or functional polarization of reactive astrocytes associated with neuroinflammation.

Systemic inflammation is reported to exacerbate pre-existing conditions and accelerate cognitive decline in several models of neurodegeneration including traumatic brain injury and prion disease [52, 53]. We acknowledge that the mice used in this study are young healthy adults. Future studies to investigate effects of DMF on systemic inflammation superimposed upon neurodegenerative conditions are warranted. Recent studies identify that Nrf2 deficiency exacerbates cognitive deficits and pathological features in mouse models of Alzheimer’s disease [54–56]. Loss of Nrf2 in the mouse brain is found to recapitulate the dysregulated pathways in human aging and Alzheimer’s disease using the transcriptomic analysis. In line with our findings of cognitive impairment in *Nrf2*^*−/−*^ mice, the long-term potentiation is found decreased in the hippocampus of *Nrf2*^*−/−*^ mice. Although the dosing regimen we selected to treat LPS-challenged *Nrf2*^*−/−*^ mice is not able to fully rescue the memory deficits, our results reveal a critical role of Nrf2 in the cognitive function and a potential of DMF as a therapeutic strategy to fight Alzheimer’s disease.

## Conclusions

The present study demonstrates that DMF attenuates microglial responses towards the inflammatory phenotype induced by LPS stimulation. The DMF treatment alleviates the acute microglial activation, neuroinflammation, long-term memory deficits, and responses of reactive astrocytes in mice following systemic immune challenge. The DMF treatment has the translational potential to protect neurons against toxic microenvironments in the brain associated with systemic inflammation.

## Additional files

Additional file 1: **Figure S1.** Characterization of microglial cultures, cell toxicity of DMF, and enriched astrocyte cultures. (PDF 134 kb)

Additional file 2: **Figure S2.** Inflammatory activation of MG induced by LPS. (PDF 146 kb)

Additional file 3: **Figure S3.** Cognitive assessment following systemic immune challenge and DMF treatment. (PDF 154 kb)
