# Supplementary material for: Dimethyl fumarate attenuates reactive microglia and long-term memory deficits following systemic immune challenge
Source: J Neuroinflammation. 2018 Mar 29;15:100. doi: 10.1186/s12974-018-1125-5 (PMC5877396; doi:10.1186/s12974-018-1125-5)
Supplement: Supplementary file 1 — Figure S1. Characterization of microglial cultures, cell toxicity of DMF, and enriched astrocyte cultures. (PDF 134 kb) [file 12974_2018_1125_MOESM1_ESM.pdf]

Figure S1

(A)

MG cultures

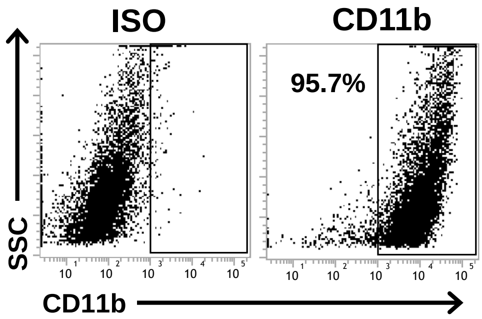

(B)

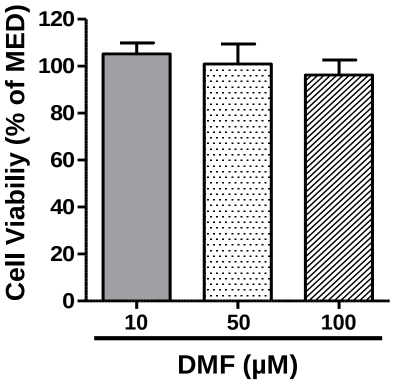

(C)

Enriched astrocyte cultures

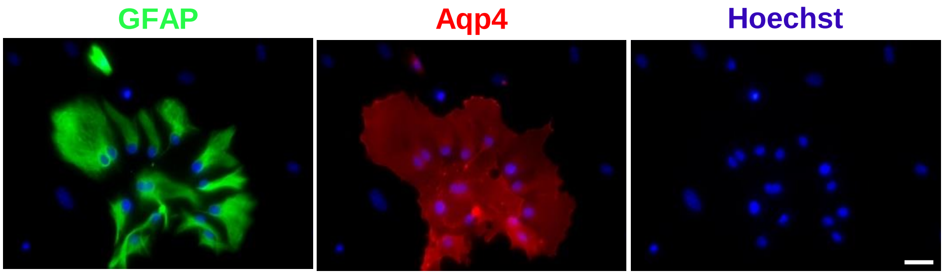

Figure S1.

Characterization of microglial cultures, cell toxicity of DMF, and enriched astrocyte cultures. (A) Purity of primary microglial cultures. Cells were harvested and analyzed on a flow cytometer to measure cell purity. Representative flow plots show that over 95% of cells express the microglial marker, CD11b. ISO, the isotype control antibody. (B) Microglial cells were incubated with DMF at different concentrations (0, 10, 50, or 100 µM) for 24h. The viability of microglial cells was examined by MTT assay. Data presented are from three independent experiments. No statistical significance was found when data were analyzed by one-way ANOVA with Bonferroni's post hoc multiple comparison test. (C) Immunocytochemical detection of GFAP (green)- and Aqp4 (red)-stained cells from enriched astrocyte cultures. Cell nuclei were stained blue with Hoechst 33342. Representative images are from three independent experiments. Scale bar, 40 µm.
