# Supplementary material for: Dimethyl fumarate attenuates reactive microglia and long-term memory deficits following systemic immune challenge
Source: J Neuroinflammation. 2018 Mar 29;15:100. doi: 10.1186/s12974-018-1125-5 (PMC5877396; doi:10.1186/s12974-018-1125-5)
Supplement: Supplementary file 2 — Figure S2. Inflammatory activation of MG induced by LPS. (PDF 146 kb) [file 12974_2018_1125_MOESM2_ESM.pdf]

**Figure S2**

**(A)**

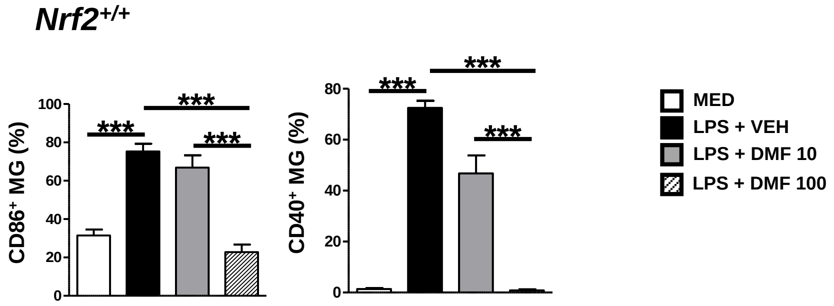


**(B)**

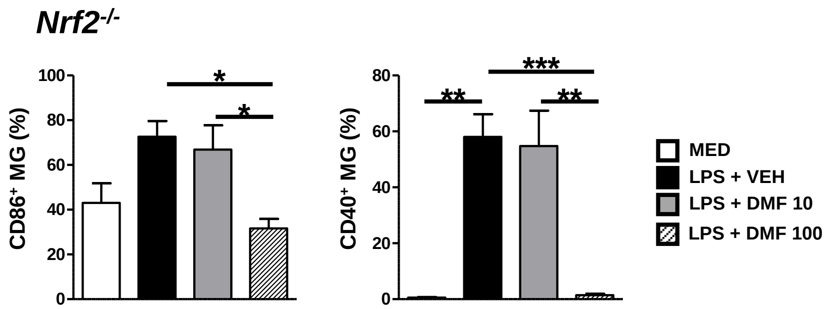


**(C)**

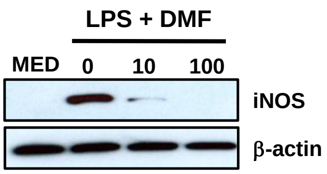

**Figure S2.**

**Inflammatory activation of MG induced by LPS.**

(A) The percentage of $CD86^+$ and $CD40^+$ cells in *Nrf2*$^{+/+}$ MG was measured in each treatment group. Data presented are from five independent experiments. ***$p < 0.001$ by one-way ANOVA with Bonferroni's post hoc multiple comparison test. MED, cell culture medium. (B) The percentage of $CD86^+$ and $CD40^+$ cells in *Nrf2*$^{-/-}$ MG was measured in each treatment group. Data presented are from three independent experiments. *$p<0.05$, **$p < 0.01$, ***$p < 0.001$ by one-way ANOVA with Bonferroni's post hoc multiple comparison test. (C) Representative western blot analysis shows that iNOS protein levels were upregulated by LPS (1 µg/ml) stimulation in MG. The cell lysates were prepared immediately following CM collected. DMF treatment suppressed iNOS protein induced by LPS stimulation in MG. Results are from three independent experiments.
