# Supplementary material for: Dimethyl fumarate attenuates reactive microglia and long-term memory deficits following systemic immune challenge
Source: J Neuroinflammation. 2018 Mar 29;15:100. doi: 10.1186/s12974-018-1125-5 (PMC5877396; doi:10.1186/s12974-018-1125-5)
Supplement: Supplementary file 3 — Figure S3. Cognitive assessment following systemic immune challenge and DMF treatment. (PDF 154 kb) [file 12974_2018_1125_MOESM3_ESM.pdf]

Figure S3

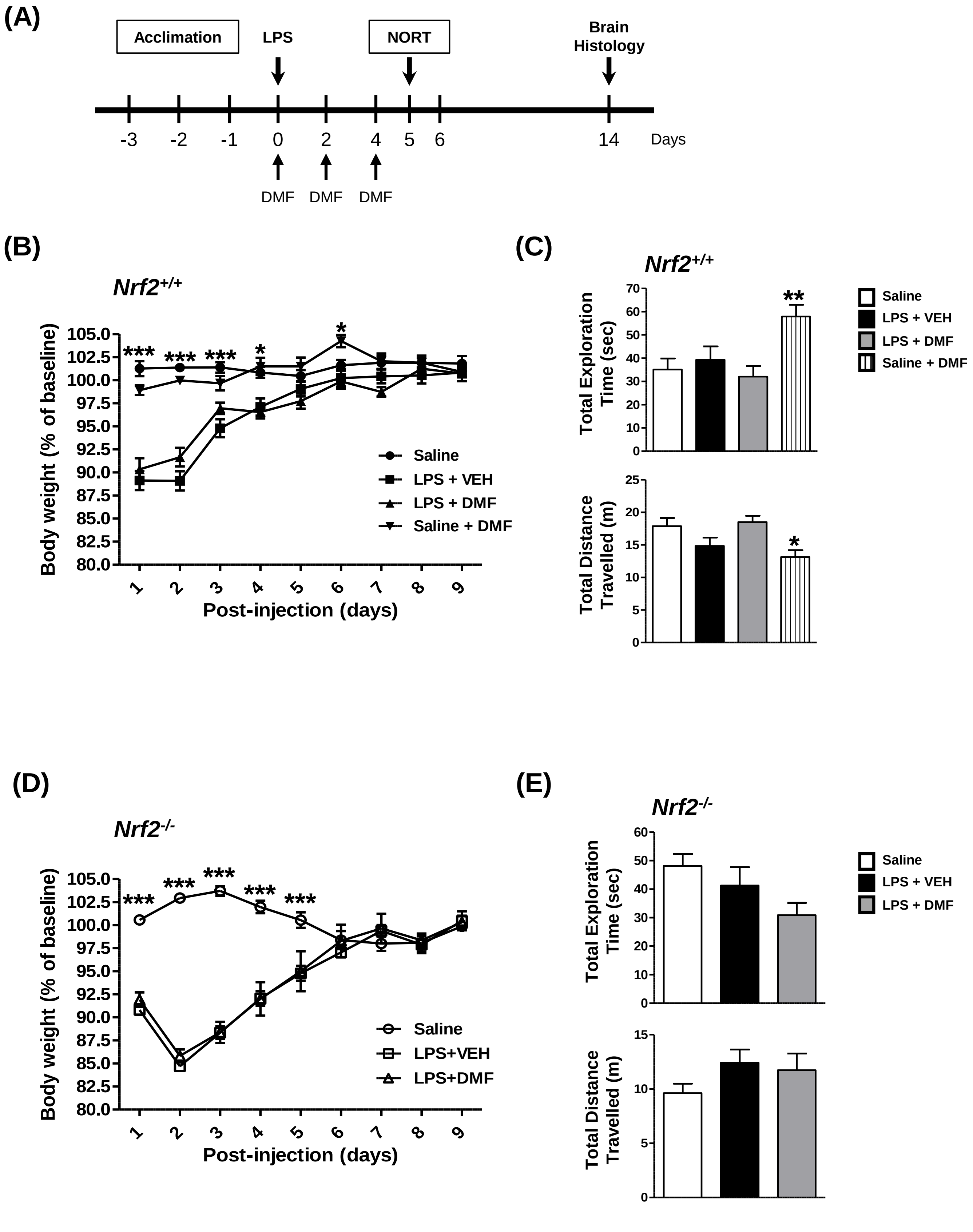
(A)
Acclimation
LPS
NORT
Brain Histology
-3 -2 -1 0 2 4 5 6 14 Days
DMF DMF DMF
(B)
Nrf2+/+
Body weight (% of baseline)
105.0 102.5 100.0 97.5 95.0 92.5 90.0 87.5 85.0 82.5 80.0
*** *** *** * *
Saline
LPS + VEH
LPS + DMF
Saline + DMF
1 2 3 4 5 6 7 8 9
Post-injection (days)
(C)
Nrf2+/+
Total Exploration Time (sec)
**
Total Distance Travelled (m)
*
Saline
LPS + VEH
LPS + DMF
Saline + DMF
(D)
Nrf2-/-
Body weight (% of baseline)
*** *** *** *** ***
Saline
LPS+VEH
LPS+DMF
Post-injection (days)
(E)
Nrf2-/-
Total Exploration Time (sec)
Total Distance Travelled (m)
Saline
LPS + VEH
LPS + DMF

Figure S3.

Cognitive assessment following systemic immune challenge and DMF treatment.

(A) Experimental timeline of cognitive assessment in mice that received LPS injection. Mice were acclimated to the apparatus for three days before LPS (1 mg/Kg, ip) injection. DMF (30 mg/Kg, ip) was administrated at day 0, 2, and 4 post-LPS as indicated. The novel object recognition task (NORT) was performed at day 5 post-LPS as indicated. The brains were harvested 14 days after LPS challenge for histology examinations. (B) The body weight of *Nrf2*$^{+/+}$ mice (C57BL/6) that received saline, LPS + VEH, LPS + DMF, or saline + DMF treatment was measured every day after LPS injection. The body weight before injection from each mouse was used as the baseline. Data presented are changes of body weight from the baseline. N = 10-16 mice/group. *$p<0.05$, ***$p<0.001$ by two-way repeated measures ANOVA with Bonferroni's post hoc multiple comparison test (LPS+ VEH v.s. saline; LPS + DMF v.s. saline). There was no statistical significance between saline and saline + DMF treatment groups. (C) The total exploration time and distance travelled of each mouse during the recognition phase of NORT were acquired. Data presented are values from four treatment groups, saline, LPS + VEH, or LPS + DMF, saline + DMF. N = 10-16 mice/group. *$p<0.05$, **$p<0.01$ by one-way ANOVA with Bonferroni's post hoc multiple comparison test. (D) The body weight of *Nrf2*$^{-/-}$ mice that received saline, LPS + VEH, or LPS + DMF injection was measured daily after LPS injection. The body weight before injection from each mouse was used as the baseline. Data presented are changes of body weight from the baseline. N = 8-12 mice/group. ***$p<0.001$ by two-way repeated measures ANOVA with Bonferroni's post hoc multiple comparison test (LPS+ VEH v.s. saline; LPS + DMF v.s. saline). (E) The total exploration time and distance travelled of each *Nrf2*$^{-/-}$ mouse during the recognition phase of NORT were acquired. Data presented are values from three treatment groups.
